# Proportional assist ventilation versus pressure support ventilation for weaning from mechanical ventilation in adults: weaning success and mortality

**DOI:** 10.1186/s13054-021-03575-9

**Published:** 2021-06-10

**Authors:** Ioannis Pantazopoulos, Georgios Mavrovounis, Maria Mermiri, Christos Kampolis

**Affiliations:** 1 Department of Emergency Medicine, Faculty of Medicine, School of Health Sciences, University of Thessaly, 41110 Larissa, Greece; 2 Department of Anesthesiology, Faculty of Medicine, School of Health Sciences, University of Thessaly, Larissa, Greece; 3 Department of Emergency Medicine, Ippokrateio General Hospital, Athens, Greece

**To the Editor,**

We have read with great interest the timely and well written meta-analysis by Ou-Yang et al. [1]. The authors conclude that proportional assist ventilation (PAV), when used as a weaning method, improves the weaning success rate. Although we agree with the main conclusion of the authors, we would like to comment on some of the statements that were made.

Firstly, in the Materials and Methods section, the authors state that “The primary outcome was weaning success, defined as the absence of the requirement for invasive mechanical ventilation support…”. Nevertheless, in the analysis of weaning success, we noted that the authors have included patients from the study by Xirouchaki et al. [2] that remained on proportional assist ventilation with load adjustable gain factors (PAV +) for 48 h. According to the definition stated by Ou-Yang et al. [1], switching from a controlled mode to assist ventilation should not be considered as weaning success. Figure 1 depicts the meta-analysis of weaning success with the proposed change. PAV is still associated with a significantly higher rate of weaning success but with a risk ratio of 1.20 (95% CI 1.07, 1.34) compared to 1.16 (95% CI 1.07–1.26) that was reported by the authors.

**Fig. 1:**
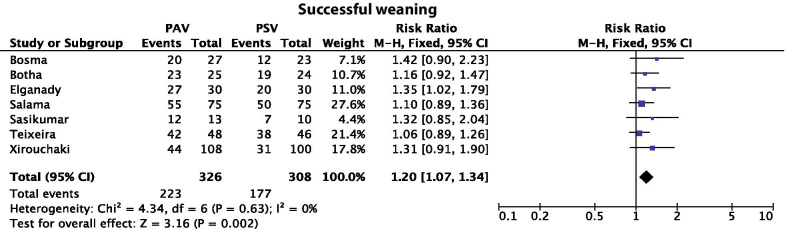
Meta-analysis of weaning success. *PAV* proportional assisted ventilation, *PSV* pressure support ventilation, *CI* confidence interval

Furthermore, the authors state that one of the secondary outcomes that was investigated is in-hospital mortality. However, with the exception of the study by Elganady et al. [3], the numbers used in the analysis for in-hospital mortality correspond to the numbers for ICU mortality. In order to clarify this, Fig. 2 presents the results of meta-analyses for both in-hospital mortality and ICU mortality between PAV and pressure support ventilation (PSV).

**Fig. 2:**
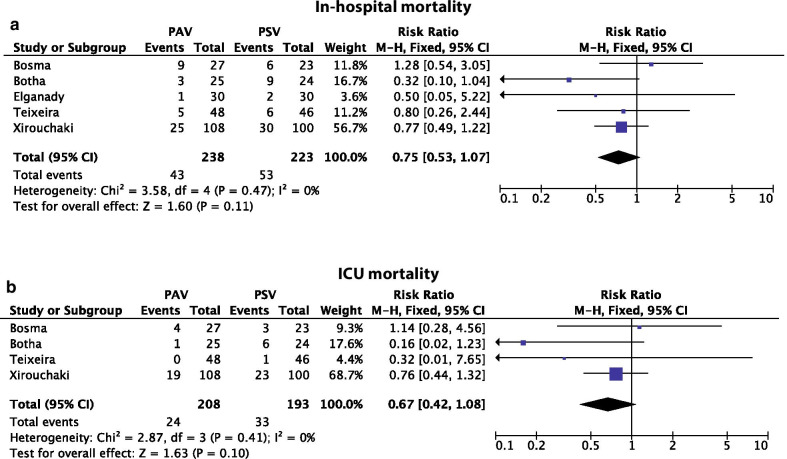
Meta-analyses of **a** in-hospital, **b** ICU mortality. *PAV*, proportional assisted ventilation, *PSV* pressure support ventilation, *CI* confidence interval

In conclusion, we fully agree with the main conclusion of the well-executed systematic review and meta-analysis by Ou-Yang LJ et al., but we wanted to point out those inaccuracies in order to provide accurate and consistent information to the scientific community.

## Data Availability

All data generated or analysed during this study are included in this published article [and its supplementary information files].
