# Supplementary material for: Proportional assist ventilation versus pressure support ventilation for weaning from mechanical ventilation in adults: weaning success and mortality
Source: Crit Care. 2021 Jun 10;25:200. doi: 10.1186/s13054-021-03575-9 (PMC8194124; doi:10.1186/s13054-021-03575-9)
Supplement: Related texts

# Reply: Authors’ response

**Authors:** Liang-Jun Ou-Yang, Po-Huang Chen, Hong-Jie Jhou, Vincent Yi-Fong Su, Cho-Hao Lee

We appreciate the precise comments by Pantazopoulos et al., pointing out the details of our outcome descriptions.

According to the study by Xirouchaki et al. [2], 108 patients were included in the proportional assist ventilation (PAV) group initially, with 96 of them did not meet the “failure criteria”, which meant that switching to the controlled mode was not necessary. Among them, 27 patients were extubated successfully. In the pressure support ventilation (PSV) group, 19 patients were extubated. We carefully performed the meta-analysis again using the corrected data mentioned above, which is shown in Fig. 3. The rate of weaning success was significantly greater in patients receiving PAV compared to patient undergoing PSV (fixed effect, RR 1.19 CI 1.06–1.33, *I*^2^ = 0%, Cochran Q *p*-value: 0.67). We also utilized the corrected data to perform trial-sequential analysis of our major outcome, the weaning success. The TSA-adjusted RR was 1.19 (95% CI 1.01–1.39). The main conclusion does not alter despite the data modification.

**Fig. 3:** Forest plot of the weaning success. The event numbers of both PAV and PSV groups for Xirouchaki 2008 [2] were corrected

In our meta-analysis, five of seven included studies compared the mortality rate in both PAV and PSV group. The study by Elganady et al. [3] evaluated 28-day mortality, and other four studies evaluated both in-hospital and ICU mortality. We are very grateful that Pantazopoulos et al. helped clarifying our mistakes and performed meta-analyses of both ICU and in-hospital mortality. “All-cause mortality” may be a more proper term for the mixed data we presented initially.

In summary, the outcomes of weaning success, ICU mortality, and in-hospital mortality were double checked. According to the meta-analysis and TSA using the revised data, the PAV can still significantly improve the weaning success rate. Considering the mortality, “all-cause mortality” may be more appropriate to described the results presented in our original manuscript. We thank Pantazopoulos et al. again for their valuable comments on our meta-analysis, helping us to provide more accurate information to the academic communities.
